# Integration of a smartphone app with posttraumatic stress disorder treatment for frontline workers: a pilot study

**DOI:** 10.1080/00049530.2024.2399112

**Published:** 2024-09-09

**Authors:** Mark Deady, Daniel A. J. Collins, Suzanna Azevedo, Eileen Stech, Anthony Harrison, Catherine Broomfield, Srishti Yadav, Aimee Gayed, Samuel B. Harvey, Richard Bryant

**Affiliations:** a Black Dog Institute, Faculty of Medicine & Health, University of New South Wales, Sydney, NSW, Australia; b School of Psychology, University of New South Wales, Sydney, NSW, Australia

**Keywords:** Smartphone app, mHealth, eHealth, posttraumatic stress disorder, digital intervention, blended treatment

## Abstract

**Objective:**

Treatment of posttraumatic stress disorder (PTSD) is hindered by limited uptake, early drop-out and non-response. This pilot study aimed to explore the feasibility, acceptability, and usability of a mobile app as part of a blended approach to treating frontline workers experiencing PTSD.

**Method:**

A single-group pre-post study was conducted with 10 adult frontline workers (firefighters, police, correctional workers) receiving trauma-focused cognitive-behavioural therapy for PTSD. Participants used an app (Support Base) designed to consolidate session content and encourage independent skills practice. At post-treatment, feasibility was assessed via app usage data and participant feedback, usability via the mHealth App Usability Questionnaire, and acceptability via items from the Mobile Application Rating Scale: user version.

**Results:**

Usability ratings were above average and the app received an overall mean rating of 3.4/5. Despite generally positive attitudes towards using technology in therapy, participants had low levels of confidence/experience with wellbeing apps and almost half preferred using the standard treatment workbook. Clinicians highlighted a range of client barriers to app use, including difficulties in clinician/client collaboration. Overall, there was significant pre- to post-treatment improvement in clinical measures of PTSD and depression, but this change cannot be tied to app use.

**Conclusions:**

Although Support Base was viewed by participants as usable and acceptable, there were feasibility issues which must be further considered in delivering this form of care. Due to the uncontrolled study design, any additive effects of the app beyond standard clinical treatment could not be assessed. The development of a more integrated blended care model is a potential avenue for future research.

## Key Points

What is already known about this topic:

(1) Frontline workers are at high risk of developing PTSD along with detrimental impacts to occupational and daily life functioning.

(2) Effective PTSD treatments are available, yet both uptake and treatment response are sub-optimal.

(3) Mobile app-based interventions are a promising way to improve access, adherence, and response to treatment while saving practitioner time.

What this study adds:

(1) In this pilot study, an app-based adjunct to trauma-focused cognitive-behavioural therapy was found to be usable and acceptable for frontline workers.

(2) This type of blended care for PTSD is unlikely to suit all frontline workers in its current form.

(3) Future research should focus on a more integrated blended care model with increased capacity for client/clinician collaboration.

## Introduction

Frontline workers are routinely exposed to repeated traumatic events, placing them at increased risk of developing a range of mental health conditions, most notably posttraumatic stress disorder (PTSD) (Carleton et al., 2018; Harvey et al., 2016). Prevalence rates of PTSD are estimated at around 10% among frontline/emergency workers such as paramedics, firefighters, and police (Berger et al., 2012; Kyron et al., 2022). The condition is associated with comorbid mental disorders and suicidality, as well as significant functional and work impairment, loss of productivity, and increased risk of adverse life outcomes (Kessler, 2000).

The recommended treatment for PTSD, as recognised by most international treatment guidelines, is trauma-focused psychotherapy (Forbes et al., 2020, National Collaborating Centre for Mental Health, 2005). However, the majority of those with PTSD do not receive treatment at all (Kessler, 2000) or drop out early (Fernandez et al., 2015; Imel et al., 2013). Major reasons for not receiving or completing treatment include stigma, prohibitive costs, and limited availability of providers (National Academies of Sciences, Engineering, and Medicine, 2018; Stecker et al., 2013). Even where those in need receive treatment, the nature of treatment received may at times be inadequate, inappropriate, or non-evidence-based (Koenen et al., 2017; Lu et al., 2016; Watts et al., 2014). Furthermore, there are additional issues pertaining to non-response and lack of adherence even where appropriate evidence-based treatments are used (Loerinc et al., 2015; Schottenbauer et al., 2008). For example, avoidance of trauma-related stimuli may hinder participant engagement in treatment and contribute to dropout (Watkins et al., 2018). Relatedly, meta-analytic evidence suggests trauma-focused psychotherapy is associated with higher rates of dropout than other forms of PTSD treatment that place less emphasis on exposure-based techniques (Lewis et al., 2020).

Mobile app-based interventions are a promising way to improve access to, retention in, and successful response to mental health treatment (Lattie et al., 2022). However, there is limited evidence that standalone apps (i.e., used without professional or clinical support) are effective in treating symptoms of PTSD (Goreis et al., 2020; Wickersham et al., 2019). Nevertheless, an app designed to target PTSD in military veterans has shown greater effectiveness when combined with clinician support than when used as a standalone tool (Possemato et al., 2016). Moreover, studies of treatment for depression, anxiety and other mental health disorders suggest that a blended care approach (combining face-to-face and internet-based components) may result in similar outcomes to face-to-face treatment, while also reducing clinician time (Erbe et al., 2017; Ly et al., 2015). Australian national data suggests that appropriate use of a blended face-to-face and digital care model would result in considerable savings to practitioner time, thereby increasing capacity to see more clients, cutting wait times, and alleviating health system burden (Australian Bureau of Statistics, 2022). There is also some evidence from meta-analysis of psychological and behavioural interventions that mobile-supported treatment can result in increased therapeutic benefit compared to face-to-face care alone, corresponding to a small effect size (ES = 0.27) (Lindhiem et al., 2015). The use of an app in combination with clinical treatment poses several potential advantages (particularly regarding between-session homework tasks), such as more engaging activities, automated reminders to complete tasks, and accountability and support via sharing of progress with therapist. These features may in turn counter avoidance and improve homework completion, which is associated with better treatment outcomes (Cooper et al., 2017). However, there have been few studies to date assessing apps designed to support or complement face-to-face treatment for PTSD specifically.

Through a co-design process involving frontline workers, clinicians, and technical experts in digital mental health, the current research team has developed an app intended to be used by frontline workers to support face-to-face therapy sessions, as part of a blended approach to treating PTSD (Deady et al., 2023). Within a standardised framework for creating mobile mental health apps, pilot testing is recommended to evaluate and assess the usability and acceptability of the app (Schellong et al., 2019).

This pilot evaluation will allow us to consider the practical utility of a smartphone app-based intervention as part of a blended care approach to treating frontline workers with established psychological problems, particularly PTSD. The aim of this study is to explore the feasibility, acceptability, and usability of a mobile app designed to support treatment for frontline workers experiencing PTSD. Information gained from this critical process will help inform further app development and improvements prior to more rigorous evaluation.

## Methods

### Study design

A single-group pilot study was conducted in Australia within a research clinic specialising in PTSD treatment. Frontline workers attending the clinic were invited by their clinicians to use the app to support their treatment sessions. Participants completed a series of evaluation measures before and after treatment.

The study was approved by the University of New South Wales (UNSW) Human Research Ethics Committee (HC210528). Participants were undertaking treatment via trial (HC210804; ACTRN12621001442897). All procedures were carried out in accordance with the Declaration of Helsinki (World Medical Association, 2013).

### Participants

Participants were required to be: (1) aged 18 or older; (2) a serving or retired first responder or frontline industry worker (e.g., fire and rescue, police, ambulance, corrective services); (3) receiving PTSD treatment (either face-to-face or telehealth) via the participating research clinic; (4) currently residing in Australia; with (5) adequate comprehension of English. Individuals were excluded from the study if they did not own a compatible smartphone.

### Procedures

Participants were recruited from May to October 2022 on a volunteer basis. Clients attending the PTSD research clinic for face-to-face or telehealth treatment who met the study eligibility criteria (and had attended ≤4 sessions of treatment) were provided with an advertisement outlining the purpose of the study. Participation in this study did not affect their access to clinical treatment, which occurred regardless of involvement in the study. Clients interested in participating notified their treating clinician, who provided them with a participant information statement and consent form. They were required to return a signed consent form to indicate informed consent. The participant’s first name and email address were then uploaded to the online trial management system, and an automatically generated email was sent to the participant including a link to complete an online baseline assessment. They received up to two reminder emails if they did not complete the survey within 1 week. Upon completion of the baseline, they were provided with access to the app via an automated email, including a unique login code and instructions to download the app from the App Store/Google Play Store.

At the end of treatment (12 weeks post-baseline, or earlier if the participant completed the baseline partway through treatment), an email invitation was sent to participants with a link to complete the online post-treatment assessment. They received up to two reminder emails if they did not complete the assessment within 2 weeks of receiving it.

#### Smartphone application

*Support Base* is a smartphone app designed to support clinical treatment of PTSD in frontline worker populations. It was developed through an iterative process involving both frontline workers who had previously received treatment and clinicians specialising in PTSD, along with input from workplace mental health researchers, user experience experts, digital learning specialists and app developers (Deady et al., 2023). In line with co-design findings, the app content was designed to support completion of homework tasks and reinforce key concepts between face-to-face sessions. *Support Base* underwent considerable user testing during development, including focus testing of the app prototype with corrective service workers (i.e., frontline staff with high rates of trauma exposure) who rated the app highly in terms of usability (>80/100 on the System Usability Scale).

**Figure 1.**
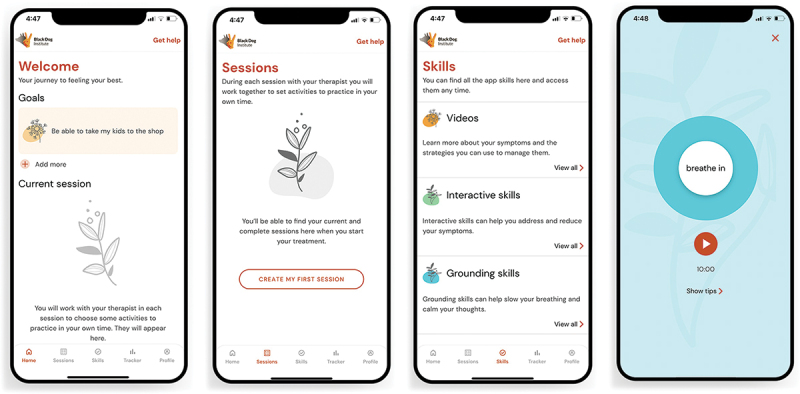
Screenshots of the Support Base app.

The core app content included psychoeducation videos, interactive cognitive and exposure tasks, animations to encourage grounding skills, and mindfulness audio exercises (see Figure 1). Participants in this study were instructed to use the *Support Base* app for the duration of their clinical treatment (as app onboarding took place within the first 1 to 4 sessions, length of usage varied). Therapists received an app introduction session presented by the research team members who developed the *Support Base* app, along with a user guide for reference during the trial (this was not shared with clients). With guidance from their therapist, participants were encouraged to complete relevant homework tasks each week within the app to consolidate session content and encourage skills practice between sessions. They could also export a summary of completed homework tasks and download this for their own records or send to their therapist. Additional features included a goal-setting function, activity reminders, links to crisis support services, and a coping plan to mitigate post-treatment relapse.

#### Clinical treatment

Participants concurrently received trauma-focused CBT, the gold standard treatment for PTSD as recommended by international treatment guidelines (Forbes et al., 2020; National Collaborating Centre for Mental Health, 2005). The manualised treatment programme was designed specifically for emergency service workers and delivered over 12 weekly sessions (of 1.5 hours in length), either face-to-face or via videoconferencing (Bryant et al., 2019). The program included psychoeducation, imaginal reliving of trauma memories, cognitive challenging, in-vivo exposure to avoided situations, coping skills (for managing associated problems such as depression, anger, guilt and shame, substance use, sleep problems, physical pain) and relapse prevention. The treatment programme was not evaluated in the present study.

Participants were also provided with a hardcopy client workbook during treatment, as per standard clinic procedures. The workbook was used both within and between therapy sessions. The workbook was the primary source for content included in the *Support Base* app and contained information to consolidate treatment session content and encourage skills practice. Participants could elect to use the workbook in tandem with app usage.

### Outcome measures

The online baseline assessment collected data on participant demographics (age, gender, education) occupation (employment status, current/former frontline service, number of years as a frontline worker), smartphone use (type of phone, frequency of use), and experience of wellbeing apps. Nine items were included to assess attitudes towards use of technology in therapy, based on the Unified Theory of Acceptance and Use of Technology (UTAUT) model (e.g., see (Heerink et al., 2009; Venkatesh et al., 2003)). Scores on these items were measured on a 5-point Likert scale, with total scores ranging from 9 to 45 (higher scores indicate more positive attitudes). At post-treatment assessment, questions on attitudes towards the use of technology in therapy were repeated.

#### Feasibility

Feasibility of the blended approach was primarily based on app usage and engagement data. This involved objective and self-report measures. Objective use data was collected automatically and stored within the online trial management system. This included time spent in app, specific activities accessed, completed activities, and use of export homework function. Additionally, participants were asked a range of questions to capture subjective app usage. These items ascertained the stage of treatment at which app was downloaded, average weekly app usage and hardcopy workbook usage.

#### Usability and acceptability

##### mHealth App Usability Questionnaire (MAUQ)

The primary usability measure was the MAUQ for standalone mental health apps (patient version), an 18-item scale intended specifically for evaluating usability of mHealth applications (Zhou et al., 2019). The MAUQ has strong construct and criterion validity, and three subscales with high internal consistency: ease of use, interface/satisfaction, and usefulness (Zhou et al., 2019). Each item is positively worded and measured on a 7-point Likert scale, with total scores ranging from 18 to 126 (higher scores indicate better usability).

##### Mobile Application Rating Scale: user version (uMARS)

Four items were included from the uMARS (Stoyanov et al., 2016) subjective quality subscale: (1) likelihood of recommending the app to others, (2) expected usage over the next 12 months, (3) likelihood of paying for the app, and (4) overall star rating. Scores for each item ranged from 1 (lowest rating; e.g., “One of the worst apps I’ve used”) to 5 (highest rating; e.g., “One of the best apps I’ve used”).

##### Overall feedback

Feedback was collected from participants specifically around app versus. workbook preference, suggestions for additional app features and open feedback/comments. Clinicians provided written feedback on their experiences in using the app with clients, and participated in a group discussion session at closure of the trial.

#### Mental health outcomes

As this study was conducted during clinical treatment, mental health outcome data were also collected. Although no true effectiveness could be examined, we have reported the pre- and post-treatment mental health outcomes of the current sample in order to determine any unintended negative effects associated with the app-augmented protocol and monitor within-group patterns of clinical recovery and symptomatology. Within-group (uncontrolled) change was explored over time regardless of app usage.

##### Posttraumatic stress disorder

Clinician-measured PTSD symptom severity was assessed using the Clinician-Administered PTSD Scale for DSM-5 (CAPS-5) (Weathers, Blake, et al., 2013) and self-reported symptomatology was measured by the PTSD Checklist for DSM-5 (PCL-5) (Weathers, Litz, et al., 2013). Past month symptoms were rated prior to treatment and past week symptoms were rated post-treatment.

The CAPS-5 is a gold standard, structured diagnostic interview for PTSD conducted by a clinically trained assessor, and is designed to measure DSM-5 PTSD symptoms (Weathers, Blake, et al., 2013). The CAPS-5 comprises 20 questions, each scored on a 5-point Likert scale that indexes symptom severity (0 = *none*, 4 = *extreme*). These are summed to provide an overall severity score (range 0–80; higher scores indicate greater severity).

The PCL-5 is a 20-item self-report questionnaire reflecting the DSM-5 PTSD criteria (Weathers, Litz, et al., 2013). Psychometrically robust with strong reliability and validity, the PCL-5 has been validated in a range of populations (Blevins et al., 2015; Wortmann et al., 2016).

##### Cognitions

Trauma-related thoughts and beliefs were measured using the Posttraumatic Cognitions Inventory (PTCI (Foa et al., 1999)). The PTCI has strong construct validity with established measures and the ability to discriminate well between traumatised individuals with and without PTSD (Foa et al., 1999).

##### Depression symptoms

The Beck Depression Inventory (2nd ed.; BDI-II) is a 21-item self-report inventory designed to measure depressive symptoms in “the past 2 weeks” (Beck et al., 1996). Items are scored on a 4-point Likert scale and summed to provide an overall severity score (range 0–63; higher scores indicate greater severity). The BDI-II has excellent reliability (*α* = 0.92) and test – retest reliability (*r* = 0.90) (Beck et al., 1996).

#### Statistical analysis

All data were analysed in IBM SPSS Statistics v27.0 (SPSS Inc, Chicago, IL, USA). Descriptive statistics were used to explore mean usability and acceptability scores. Post-treatment data (MAUQ score, uMARS items, and app usage/feedback) were assessed to address the primary research questions regarding app feasibility, acceptability, and usability. Two-tailed paired samples t-tests were conducted to compare pre- and post-intervention group means for the perceived acceptability of using technology in therapy and for mental health outcomes. Within-group effect size estimates of pre-post change in mental health outcomes were calculated using Hedges *g*, as this statistic allows for correction of bias due to small sample size (Lakens, 2013).

#### Sample size

The projected sample size was 10–15 participants. This was seen as appropriate to gain sufficient data to meet the research aims and answer the research questions regarding the use of the app among frontline workers undergoing treatment (this study did not intend to generalise to broader populations). Similar sample sizes have been used in other pilot studies of PTSD-related apps to ascertain feasibility, acceptability and usability (e.g., (Cernvall et al., 2018; Possemato et al., 2016)).

## Results

### Demographics and baseline measures

Twelve individuals were approached for inclusion; 11 individuals agreed to participate. One participant did not download the app and was therefore excluded from the study. Nine of the 10 participants completed the post-treatment survey (90% completion rate).

The sample (*n* = 10) predominantly identified as male (*n* = 9). The mean age of participants was 51 years (range 34–69; SD = 11.7; Table 1). Participants were employed as firefighters (*n* = 5), corrective service workers (*n* = 3), or police (*n* = 2). The majority (*n* = 7) were currently serving, while one was on medical leave and two had retired or resigned/been discharged. On average, participants had been employed as a first responder or frontline worker for 25.2 years (range 9–41; SD = 10.0).

**Table 1.** Participant demographics.

Baseline characteristic	Total sample (*N* = 10)
Age, mean (SD)	50.5 (11.7)
Gender, n (%)	
Male	9 (90.0)
Education, n (%)	
Secondary school up to Grade 12	1 (10.0)
Certificate (trade, apprenticeship, technician, etc.)	4 (40.0)
Diploma (associate, undergraduate)	3 (30.0)
Postgraduate qualification	2 (20.0)
Service (current or former), n (%)	
Police officer	2 (20.0)
Firefighter	5 (50.0)
Corrective services	3 (30.0)
Employment status, n (%)	
Currently serving	7 (70.0)
Discharged/retired/resigned	2 (20.0)
Leave/awaiting medical leave	1 (10.0)
Years of employment, mean (SD)	25.2 (10.0)

All participants owned a smartphone; seven were Apple/iOS users versus three Android users. Most (*n* = 8) used their phone one or more times per hour, while a minority (*n* = 2) used their phone only a few times a day. More than half (*n* = 6) had never used a smartphone app to support their wellbeing, although three reported they used such apps a few times a month and one participant was a daily user. At the commencement of the trial, only six users claimed they were confident using wellbeing apps.

Pre-treatment (*n* = 10) attitudes towards using technology in the therapy context were generally positive, with a mean score of 34.2 (range 31.0–37.0; SD = 2.0) out of a possible 45 across the nine items. Post-treatment (*n* = 9) ratings of attitudes towards use of technology in therapy showed a mean of 33.4 (range 24.0–40.0; SD = 5.1) across items. There was no significant difference between pre- and post-treatment attitudes overall (*t*_*8*_ = −0.35, *p* = 0.733). Individual items also showed no significant change over time (see Supplementary Table 1).

### Feasibility

Objective data on app engagement was obtained for all 10 participants via in-app data collection. In terms of the core app content, participants accessed on average 13.1 (SD = 9.47) and completed on average 8.2 (SD = 5.33) non-unique activities. Each participant accessed at least one psychoeducational video and one interactive (cognitive or exposure) activity. The most frequently viewed videos were Imaginal Reliving and Understanding Emotions (both accessed by six participants). Thought Challenging was the most frequently used interactive activity (accessed by seven participants). Five participants used the grounding skills animations, designed to train breathing and attention. The mindfulness audio recordings were not used.

The goal setting function was used by all participants. Four participants used the export homework function, which allowed them to export a summary of their app progress for personal records or to share with their therapist. In addition, four participants opted to use in-app activity reminders.

In the post-treatment survey (*n* = 9), most participants (*n* = 8) reported that they downloaded the *Support Base* app within the first three treatment sessions, while one participant downloaded the app past the halfway point of their treatment. Four participants stated they used the app for an average of <15 minutes per week, a further four used it for 15–30 minutes, and one participant used it for 1–2 hours. Workbook usage was reported to be more frequent: four participants used the workbook for an average of 15–30 minutes per week, three used it for 30–60 minutes, and two used it for 1–2 hours.

### Usability and acceptability

The mean MAUQ total score (*n* = 9) was 84.6/126 (range 42–111; SD = 25.6), with consistent scores across the subscales (ease of use = 4.73/7, SD = 1.38; interface/satisfaction = 4.71/7, SD = 1.43; usefulness = 4.65/7, SD = 1.53). The full list of scores is presented in Table 2.

**Table 2.** mHealth App Usability Questionnaire (MAUQ) scores (*n* = 9).

	Mean score (SD)	Range^a^
1. The app was easy to use.	4.56 (1.59)	2 – 6
2. It was easy for me to learn to use the app.	4.78 (1.48)	2 – 6
3. The navigation was consistent when moving between screens.	4.89 (1.36)	2 – 6
4. The interface of the app allowed me to use all the functions (such as entering information, responding to reminders, viewing information) offered by the app.	4.78 (1.39)	2 – 6
5. Whenever I made a mistake using the app, I could recover easily and quickly.	4.67 (1.50)	2 – 6
6. I like the interface of the app.	4.33 (1.87)	1 – 6
7. The information in the app was well organized, so I could easily find the information I needed.	4.89 (1.17)	3 – 6
8. The app adequately acknowledged and provided information to let me know the progress of my action.	5.00 (1.41)	3 – 7
9. I feel comfortable using this app in social settings.	4.33 (1.50)	2 – 6
10. The amount of time involved in using this app has been fitting for me.	5.11 (1.17)	4 – 7
11. I would use this app again.	4.67 (2.12)	1 – 7
12. Overall, I am satisfied with this app.	4.67 (1.80)	1 – 7
13. The app would be useful for my health and well-being.	5.11 (1.27)	3 – 7
14. The app improved my access to healthcare services.	4.22 (2.11)	1 – 7
15. The app helped me manage my health effectively.	4.67 (1.73)	2 – 7
16. This app has all the functions and capabilities I expected it to have.	4.89 (1.54)	2 – 7
17. I could use the app even when the Internet connection was poor or not available.	4.44 (1.42)	2 – 6
18. This mHealth app provides an acceptable way to receive healthcare services, such as accessing educational materials, tracking my own activities, and performing self-assessment.	4.56 (1.67)	2 – 6
Total score	84.56 (25.61)	42 – 111

^a^Range of participant scores. Possible range for each item is 1 to 7; possible range for total score is 18 to 126 (higher scores indicate better usability).

In terms of acceptability (uMARS subjective quality subscale; *n* = 9), most users (*n* = 8) reported they would recommend the app to others. Two-thirds (*n* = 6) thought they would use the app a moderate amount (3–10 times) in the next 12 months (most elements of the app can be used post-treatment completion), one reported they would use it frequently (>50 times) and the remaining two participants reported they would use it <3 times. Two participants stated they would not pay for the app, three were unlikely to pay, and four were uncertain. On average, participants gave the *Support Base* app an overall rating of 3.4/5 (range 2–5).

### User feedback

Of the nine participants who completed the post-treatment survey, only one preferred using the app over the workbook, stating it was “Easier to navigate and more engaging”. Four participants favoured the workbook, with reported reasons including personal preference for hard copy format, simplicity of workbook, avoidance of screen time, and need for a “clear disconnect from the world” to focus on treatment. The remaining four expressed no preference between the app and workbook.

In terms of additional app features, one user suggested including an alert for breathing exercises to notify the user when the exercise is finished. Another participant commented on the importance of having access to both the app and the workbook.

### Mental health outcomes

**Figure 2.**
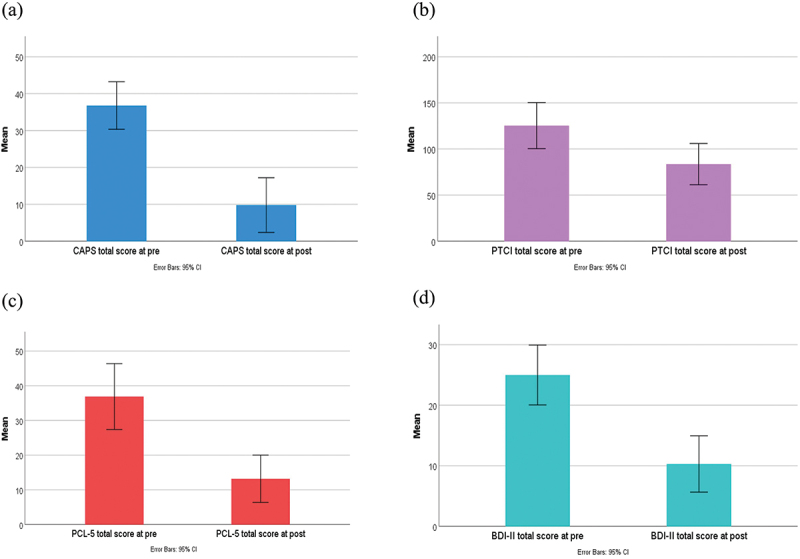
Mental health outcome mean change over time (error bars represent 95% confidence intervals). (a) CAPS: clinician-administered PTSD scale for DSM-5. (b) PTCI: posttraumatic cognitions inventory. (c) PCL-5: PTSD checklist for DSM-5. (d) BDI-ii: beck depression inventory – second edition.

All participants met the criteria for PTSD at baseline on the CAPS-5. During follow-up, only one participant remained with clinical levels of PTSD symptoms. There was a significant pre-post change on all mental health outcomes within the sample (presented in Figure 2). Mean change in CAPS-5 total score was 27 points (95% CI: 19.39–34.61; Hedge’s g = 2.54 (95% CI: 1.17–3.67)) with significant change over time (*t*_*9*_ = 8.03, *p* < 0.001). Similarly, the mean change in PCL-5 was 23.7 points (95% CI: 12.93–34.47; Hedge’s g = 1.51 (95% CI: 0.58–2.40)) with significant change over time (*t*_*9*_ = 4.98, *p* = 0.001). Mean change in BDI-II was 14.7 points (95% CI: 6.56–22.84; Hedge’s g = 1.24 (95% CI: 0.40–2.04)) with significant change over time (*t*_*9*_ = 4.09, *p* = 0.003). Mean change in PTCI was 41.8 points (95% CI: 15.62–67.98; Hedge’s g = 1.09 (95% CI: 0.30–1.85)) with significant change over time (*t*_*9*_ = 3.61, *p* = 0.006).

### Clinician reflections

Discussion and feedback from clinicians (*n* = 4) post-trial highlighted several critical considerations. Barriers to use included limited technical abilities (predominantly those of the client), client efforts to reduce phone use in daily life, some navigational issues which required simplification, and time taken in sessions to assign tasks within the app. Consistently, the major issue raised was the inability to effectively collaborate with clients, whereas pen-paper methods allowed for this and facilitated greater flexibility. Consequently, the app was less useful for some components of therapy. The psychoeducation videos, grounding tasks, and reminders were seen as the most valuable aspects of the app. Clinicians provided recommendations to simplify the app experience and integrate it with the workbook. These included: (1) the removal of the “sessions” section (clinicians suggested adding assigned tasks to the home screen without creating individual sessions, as in practice these tasks were often assigned over several sessions), (2) allowing in-vivo exposure tasks to be completed independently of the exposure ladder developed within the app, (3) incorporating alcohol use or specific symptoms into the tracking section of the app, and (4) ideally a clinician dashboard (accessible via a web portal or the clinician’s mobile device) to assign content and monitor progress. Clinical reflections praised the mindfulness section, despite no mindfulness tasks being assigned to clients during the study. They also highlighted that the app would be beneficial to specific demographics of client (younger, non-frontline worker, subclinical) or particular stages of recovery (e.g., relapse prevention following treatment).

## Discussion

This pilot study aimed to explore the feasibility, acceptability, and usability of a smartphone app designed to support trauma-focused psychotherapy for PTSD in frontline workers. Despite relatively strong usability and acceptability ratings, there were issues related to feasibility which need to be addressed in future versions of the app. The most favoured components were those which were highly visual and interactive but did not include text entry, such as psychoeducation videos and animations designed to train grounding skills. Primarily, the use of the app as a tool for setting and completing homework tasks was less collaborative and flexible than manual versions of task completion and therapist interaction. Changes to both the app and the protocol for integrating the app into treatment are required. Similarly, there was a disinclination to use the app for many workbook activities.

All participants used the *Support Base* app during their treatment and accessed a range of features and activities. All participants accessed the psychoeducation videos, interactive skills activities and goal-setting function, while half used the app’s grounding exercises. Mindfulness was an optional task assigned at the therapist’s discretion and it appears that (despite clinician praise for the design) the mindfulness audio recordings were seldom assigned and thus not used. This highlights the integral role of the treating clinician in promoting and incorporating different modalities into blended care.

The uMARS subjective quality ratings suggest the app is acceptable for use by frontline workers in tandem with psychotherapy for PTSD. Overall, the app received an above-average rating (3.4/5), with most users stating they would recommend it to others and would continue to use the app at least a moderate amount in future, although participants generally stated they would not pay for the app. These results are comparable with results from studies testing other apps designed as adjunctive therapy or self-management tools for PTSD in Australia (Shakespeare-Finch et al., 2020).

The validated usability measure employed in this study (MAUQ (Zhou et al., 2019)) has the advantage of being designed specifically for app user testing with clients/patients (Muro-Culebras et al., 2021). MAUQ ratings were above average for all items (Mustafa et al., 2021), providing evidence of satisfactory levels of app usability among this group. There were also no major technical issues reported by participants. The highest scores were seen for usefulness as a health/wellbeing tool, and time-efficiency (both items rated 5.1/7). The mean usefulness subscale rating of 4.65/7 is equivalent to ratings of other health apps tested in a relatively similar trauma-exposed population of military veterans (4.88/7 (Williamson et al., 2022)). Compared to this prior research, *Support Base* ease of use and interface/satisfaction did not score as highly (despite still being rated above average), suggesting that these areas could be targets of improvement in future iterations of the app. However, the lower scores on these subscales may be partly due to the complexity necessitated by the blended model of *Support Base*, which involved collaborative use of the app with a therapist, unlike the prior study by Williamson and colleagues (Williamson et al., 2022).

Despite above-average usability ratings and good indicators of app acceptability, a major barrier regarding feasibility was that of limited participant motivation to utilise digital treatment options and lack of confidence with technology. This was highlighted both by the self-reported low levels of experience/confidence with wellbeing apps at the beginning of the study and overall feedback favouring manual methods of task completion. Nearly half the sample stated they preferred using the conventional therapy workbook over the app. Reasons provided for this preference were entirely personal and not related to the app itself but included a deliberate avoidance of technology and personal preference for using hardcopy for retaining/rehearsing information. One participant also pointed out the importance of providing a choice of delivery modality. Interestingly, despite the reluctance reflected in these findings, attitudes towards using technology in therapy at baseline were relatively high. No significant change in attitudes was found after use of the app, which may have been due to ceiling effects. Given the apparent ambivalence of participants in integrating the app within treatment (despite a generally positive initial response to the idea of blended care), a key question for future research is whether therapist support and encouragement of app use can lead to greater usage and/or improved treatment outcomes.

Collectively, these findings highlight that an app-based adjunct to psychotherapy has much potential, but is unlikely to be universally appropriate despite being acceptable, usable, and fit-for-purpose (Deady et al., 2023). This programme underwent significant co-design which informed content, interface, and delivery (Deady et al., 2023). However, based on the current findings, the potential advantages afforded by an app (e.g., accessibility, flexibility, convenience, interactivity (Marcu et al., 2022)), while appealing to many participants, were not well harnessed by all. This is likely due to several factors. First, the population were highly motivated to receive treatment, as evidenced by seeking to participate in a clinical trial, and treatment was delivered in a gold standard manner. Although the app was designed to support homework completion, emergency service workers in clinical trials have already been shown to have high levels of engagement when this form of care is attained (Bryant et al., 2019). Second, demographic factors of the current sample were not consistent with that of high adopters of new technologies (Talukder, 2012). The findings among this group suggest a preference for hardcopy workbook content rather than an app, and augmentation with mobile technology may therefore be better suited to other populations experiencing PTSD. Third, this augmentation of treatment represented a major deviation from usual care for both client and clinician and thus there are likely to be some difficulties adjusting to this form of blended care. Clinician reflections highlighted important changes that could be made to the app but also how the app might be best utilised in clinical practice, such as with non-frontline worker groups, younger populations, and those who are more comfortable with digital technologies, along with suggestions for use with subclinical groups outside the blended treatment context. Additionally, prevention of relapse may be an alternative use for the app.

This study was limited to a small sample size, which did, however, include workers from a variety of frontline agencies. This sample size restricted comparison of use by different groups (i.e., participants were generally older and predominantly male). Although clinicians were trained in how to use the app, this formed a new component of care and clinicians had varying degrees of comfort in integrating the technology into sessions. There may thus have been significant variation in how the app was implemented within treatment (e.g., level of guidance or encouragement of app use), however therapist fidelity to the blended care model was not assessed. Equally, therapist factors (e.g., age, years of clinical experience, prior use of digital technology in therapy) may have impacted the delivery of the protocol but were not assessed. In addition, delivery of the app in a specialist PTSD treatment/research clinic setting may have impacted findings bidirectionally. For example, this could positively or negatively influence client expectations about care quality (specialist care might be expected to lead to symptom improvement, but involvement in experimental research might undermine this expectation). As with most studies, the nature of this controlled environment may also not generalise to all therapists or clinical settings. Nevertheless, as noted, the findings suggest that optimum use of the app was not achieved in the present clinical context. During codesign, both clients and clinicians reported a reluctance for the app to be used to reduce the number of face-to-face sessions (Deady et al., 2023). Consequently, the model for combining the app with face-to-face care tested here failed to fully encapsulate the potential benefits of a more balanced blended care approach. Future work to develop a more integrated model may hold promise but requires client and clinician training and enhanced user appeal. Finally, as evident here, implementation of any protocol embedding digital health offerings may be constrained by smartphone access, technological literacy, and inclination to use digital tools.

This pilot study highlighted some of the likely benefits as well as some feasibility issues involved in delivering a blended care offering to frontline workers seeking psychotherapy for PTSD. Although the sample rated the smartphone app relatively strongly for usability and acceptability, there were issues related to feasibility which must be considered in the delivery of this form of care. Fundamentally, an app-based adjunct to PTSD treatment may hold utility for some individuals but is unlikely to suit all treatment seekers. Determining how best to integrate technological offerings into treatment is pivotal to harnessing the potential of blended models of care.

## Data Availability

The data that support the findings of this study are available from the corresponding author, MD, upon reasonable request.
